# Comparison of commercial assays and two-step approach to detect *Clostridioides difficile* in South Africa

**DOI:** 10.4102/ajlm.v11i1.1809

**Published:** 2022-09-29

**Authors:** Sarishna Singh, Mae Newton-Foot, Pieter Nel, Colette Pienaar

**Affiliations:** 1 National Health Laboratory Service Tygerberg Academic Laboratory, Division of Medical Microbiology, Tygerberg Hospital, Tygerberg, South Africa; 2 Division of Medical Microbiology, Department of Pathology, Faculty of Medicine and Health Sciences, Stellenbosch University, Cape Town, South Africa

**Keywords:** *Clostridioides difficile*, *Clostridium difficile*, Xpert, BD MAX, QUIK CHEK, toxigenic culture

## Abstract

**Background:**

*Clostridioides difficile* is the number one cause of hospital-acquired diarrhoea. Accurate diagnosis of *C. difficile* is of utmost importance as it guides patient management and infection control practices. Studies evaluating the performance of commercially available nucleic acid amplification tests (NAATs) versus algorithms are lacking in resource-limited settings.

**Objective:**

This study assessed the performance of three commercially available tests and a two-step approach for the diagnosis of *C. difficile* infection using toxigenic culture (TC) as the gold standard.

**Methods:**

Two hundred and twenty-three non-duplicate loose stool samples were submitted to the National Health Laboratory Service Microbiology Laboratory at Tygerberg Hospital, Cape Town, South Africa, from October 2017 to October 2018. The samples were tested in parallel using the *C. DIFF QUIK CHEK COMPLETE* enzyme immunoassay (EIA) and two NAATs (Xpert *C. difficile* and BD MAX Cdiff), and the results were compared to TC. The performance of a two-step approach consisting of the *C. DIFF QUIK CHEK COMPLETE* followed by the Xpert *C. difficile* was also determined.

**Results:**

Of 223 faecal specimens tested, 37 (16.6%) were TC-positive. The sensitivity and specificity of the *C. DIFF QUIK CHEK COMPLETE* were 54.1% and 98.9%; Xpert *C. difficile*, 86.4% and 96.8%; BD MAX Cdiff, 89.2% and 96.8%; and two-step approach, 89.2% and 96.2%.

**Conclusion:**

The *C. DIFF QUIK CHEK COMPLETE*, in a two-step approach with the Xpert *C. difficile*, performed similarly to the NAATs on their own and offer advantages in terms of cost and workflow in low-resource settings.

## Introduction

*Clostridioides* (*Clostridium*) *difficile* is an anaerobic Gram-positive rod capable of forming endospores and producing toxins. *C. difficile* infection is associated with an asymptomatic carrier state, self-limiting diarrhoea, pseudomembranous colitis, and toxic megacolon which can be fatal.^1^ *C. difficile* infection accounts for up to 20% of nosocomial diarrhoea cases worldwide.^2^ In South Africa, there is very little published data regarding the incidence and prevalence of *C. difficile;* a previous study in patients with diarrhoea reported the prevalence to be 16%.^3^ In the past two decades, *C. difficile* has also emerged as a cause of community-acquired diarrhoea.^2,4^ Most *C. difficile* strains produce two major toxins, namely Toxin A, an enterotoxin, encoded by the *tcdA* gene, and Toxin B, a cytotoxin, encoded by the *tcdB* gene. *C. difficile* infection associated with increased severity and mortality has been reported in North America and Europe.^5^ The *C. difficile* strain responsible for these outbreaks, the hypervirulent 027/NAP1/BI, is characterised by a deletion in the *tcdC* gene (a negative regulator of *tcdA* and *tcdB* expression), resulting in increased production of Toxin A and Toxin B.^6^ Certain strains of *C. difficile* may produce a binary toxin, *C. difficile* transferase, encoded by *cdtA* and *cdtB*. Data regarding binary toxin conflict, with some studies suggesting its significance is unclear whereas others have shown that it may be associated with a higher mortality rate.^7,8,9^

Accurate diagnosis of *C. difficile* infection is essential as it guides patient management and infection control practices. The two diagnostic reference standards for the diagnosis of *C. difficile* are toxigenic culture (TC) and the cell culture neutralisation assay; however, these are labour-intensive and have extended turnaround times of 2–3 days.^10^

Current guidelines recommend a two-step approach: an enzyme immunoassay (EIA), followed by a molecular test to increase the diagnostic yield.^11^ This approach, compared to toxin EIA only, is much more sensitive. Algorithm-based testing is recommended to optimise the positive predicative value of laboratory results.^11^ Commercial EIAs utilise monoclonal antibodies to detect glutamate dehydrogenase (GDH), an antigen common to all *C. difficile* strains irrespective of toxin production, and Toxin A and/or Toxin B (Tox A/B). The *C. DIFF QUIK CHEK COMPLETE* EIA detects GDH as well as Tox A/B. Commercial nucleic acid amplification tests (NAATs) are usually real-time polymerase chain reaction (PCR) assays which target *tcdB*.

In this study, the performance of *C. DIFF QUIK CHEK COMPLETE* (*QUIK CHEK*) (Alere Techlab, Blacksburg, Virginia, United States) and two commercial NAATs, the Xpert *C. difficile* (Xpert) (Cepheid, Sunnyvale, California, United States) and BD MAX Cdiff (BDM) (Becton Dickinson, San Jose, California, United States) were compared to TC. The performance of a two-step approach consisting of the *QUIK CHEK* and Xpert, that is, the current standard of care (SOC) at our institution, was also evaluated.

## Methods

### Ethical considerations

Ethics approval was obtained from the Health Research Ethics Committee of Stellenbosch University, Cape Town, South Africa (reference number S17/03/064). A waiver of informed consent was obtained as no patient identifiers were published and no invasive procedures were performed as a result of this study. Only the results of the current SOC tests were reported to physicians. After the results were recorded, a study number was assigned to the specimen and all patient identifiers were removed.

### Study design

This was a prospective diagnostic test accuracy study comparing three different assays, namely the *QUIK CHEK*, Xpert and BDM, to TC (reference method) for the detection of toxigenic *C. difficile* in faecal samples. The performance of the current SOC (a two-step approach consisting of the *QUIK CHEK* and Xpert), at our institution was also compared to TC. A composite reference standard (CRS) analysis was performed to account for limitations in TC as a reference method (Supplementary Material).^12^ The CRS composite positive was defined as TC-positive or positive by two commercial assays in a TC-negative sample.

### Sample size

Tygerberg Hospital near Cape Town, South Africa, is a 1380-bed tertiary hospital which delivers specialist services to approximately half the population of the Western Cape province (total population 6.2 million). The Microbiology Laboratory of the National Health Laboratory Service at Tygerberg Hospital performs *C. difficile* testing on patient samples from Tygerberg Hospital as well as peripheral hospitals and clinics within the Tygerberg drainage area. Assuming a *C. difficile* prevalence of 16% based on previous studies, and using a 95% confidence interval with a 5% error rate on both sides, a sample size of 207 was calculated using the Open Epi sample size calculator (G Dean & KM Sullivan, Atlanta, Georgia, United States).^13^

### Sample processing

Non-duplicate loose stool samples (defined as taking the shape of the container) from adult and paediatric patients older than two years of age submitted to the National Health Laboratory Service Microbiology Laboratory at Tygerberg Hospital from October 2017 to October 2018 for routine *C. difficile* testing were tested in parallel with the four assays. None of the samples was frozen and thawed prior to testing. In the rare event that samples could not be tested on the day of collection, they were kept at 2 °C – 8 °C and processed within 48 h of collection.

The *QUIK CHEK* EIA was performed as per instructions provided by the manufacturer and read by multiple laboratory technologists as part of their routine daily work. The results were interpreted as follows: GDH-positive and Tox A/B-positive samples were regarded as positive, GDH-negative and Tox A/B-negative samples were regarded as negative, and GDH-positive and Tox A/B-negative samples were regarded as negative.

The Xpert NAAT was performed as per the instructions of the manufacturer. The test was interpreted as positive for toxigenic *C. difficile* if the cytotoxin gene (*tcdB*) was detected within the valid cycle threshold range and above the minimum endpoint setting, and as toxigenic *C. difficile* negative if the *tcdB* gene was not detected, provided the sample processing control and probe check controls met the manufacturer’s requirements. Testing was repeated on any samples with invalid or error results due to failure of the sample processing control or probe check controls.

The BDM NAAT was performed by following the instructions of the manufacturer. Test results were automatically interpreted by the BDM instrument. Positive, negative and unresolved results were based on the target’s and sample processing control’s amplification status. The test was interpreted as toxigenic *C. difficile* positive if the cytotoxin gene (*tcdB*) was detected within the valid cycle threshold range and above the minimum endpoint setting, and as toxigenic *C. difficile* negative if the *tcdB* gene was not detected, provided the sample processing and probe check controls met the manufacturer’s requirements. Indeterminate and incomplete results are obtained when the BDM system fails. Any unresolved, indeterminate and incomplete samples were repeated.

The two-step algorithm was performed as follows: The *QUIK CHEK* was done and samples that were GDH-positive and Tox A/B-positive were regarded as positive; GDH-negative and Tox A/B-negative samples were regarded as negative. Samples that were GDH-positive and Tox A/B-negative were interpreted in conjunction with the Xpert results to establish if toxin genes were present or absent.

Toxigenic culture was used as the reference method and performed by culturing *C. difficile* from stool samples on chromID *C.diff* agar (bioMérieux, Marcy l’Etoile, France), a differential and selective medium, followed by testing for the organism’s ability to produce toxin by PCR.^14,15,16^ A swab was dipped into the stool sample and inoculated onto the agar medium. The inoculum was streaked to enhance the recovery of single colonies. The plates were placed in a jar with an anaerobic sachet, AnaeroPack-Anaero (Mitsubishi Gas Chemical Company, Inc., Tokyo, Japan) and incubated at 35 ºC. After 48 h of incubation, the plates were examined for grey and black colonies; if none were present, the culture was considered negative for *C. difficile*. A multiplex PCR was performed on a streak of grey and black colonies to detect the *C. difficile*-specific *tpi* gene, as well as the *tcdA* and *tcdB* genes.^17^ Cultures that were PCR-positive for the *tpi* and *tcdB* genes were considered TC-positive, while those that were PCR-negative for the *tpi* or *tcdB* genes were considered TC-negative. In humans, the *tcdA* gene has been reported in *tcdB*-positive *C. difficile* strains only.^18^

### Statistical analysis

Sensitivity, specificity, positive predictive value (PPV) and negative predictive value (NPV) were calculated for each assay against both TC and the CRS using Epicalc 2000 version 1.00 software (Brixton Books, London, England).

## Results

A total of 223 samples were included in the study. Of these, 37 (16.6%) were TC-positive and 19/37 (51.4%) were also positive from all three commercial assays. The SOC positivity rate was 17.9% (40/223).

*QUIK CHEK* was GDH-pos in 45/223 (20.2%) samples. Twenty-two of the 45 samples (48.9%) were Tox A/B-pos, of which 20/22 (91%) were also TC-positive. Of the 23 GDH-pos samples that were Tox A/B-neg, 11 (47.8%) were TC-positive (Table 1).

**TABLE 1:** Performance characteristics of diagnostic assays in comparison with toxigenic culture on stool samples submitted to the National Health Laboratory Service Microbiology Laboratory at Tygerberg Hospital, Cape Town, South Africa, from October 2017 to October 2018.^1^

Test used	Toxigenic culture		Sensitivity		Specificity		Positive predictive value		Negative predictive value	
	Pos (*n* = 37)	Neg (*n* = 186)	%	95% CI	%	95% CI	%	95% CI	%	95% CI
***QUIK CHEK* (Toxin A & B)**										
Positive	20	2	54.1	37–70	98.9	96–100	90.9	69–98	91.5	87–95
Negative	17	184								
**Xpert**										
Positive	32	6	86.4	70–95	96.8	93–99	84.2	68–93	97.3	93–99
Negative	5	180								
**BD MAX**										
Positive	33	6	89.2	74–96	96.8	93–99	84.6	69–94	97.8	94–99
Negative	4	180								
**Algorithm (standard of care)**										
Positive	33	7	89.2	74–96	96.2	92–98	82.5	67–92	97.2	94–99
Negative	4	179								

Note: A total of 223 samples were included in the analyses.

CI, confidence interval.

Thirty-eight of the 223 stools (17%) tested positive by Xpert and 32/38 (84.2%) were confirmed as positive by TC. None of the samples was positive for the binary toxin or epidemic 027/NAP1/BI strain. Of the 185 Xpert-negative stools, 180 (97.3%) were confirmed as negative by TC, while 5/185 (2.7%) Xpert-negative samples were TC-positive (Table 1). The BDM detected 39/223 (17.5%) positive stool samples; 33/39 (84.6%) were confirmed by TC. One hundred and eighty of the 184 BDM-negative stools (97.8%) were confirmed as negative using TC, while the other 4/184 (2.2%) were TC-positive. The SOC two-step approach detected 40 positive stool samples, 33 (82.5%) of which were confirmed by TC. Of the 183 SOC-negative stool samples, 179 (97.8%) were confirmed as negative by TC (Table 1).

The *QUIK CHEK* performed poorly while the Xpert and BDM and the two-step approach had similar sensitivities, specificities, PPV and NPV when compared to TC. Results were also compared to a CRS (Supplementary Table 1).

The Xpert, BDM and two-step approach showed higher sensitivities, specificities and PPV in comparison with the CRS, but the NPV of all the assays were similar.

## Discussion

There is a lack of consensus regarding the optimal diagnostic *C. difficile* laboratory assays. High-quality evidence for the best diagnostic testing strategy is scarce and researchers rarely use either of the two accepted reference standards, that is, cell culture neutralisation assay or TC, for assessment of diagnostic accuracy, but rather their own laboratory-defined CRS criteria. In this study, we determined the diagnostic accuracy of three different commercial assays for the detection of toxigenic *C. difficile* in stools compared to both TC and a CRS to account for any limitations of the TC.

In this study, the sensivitity of the *QUIK CHEK* was 54.1%, Xpert was 86.4%, BDM was 89.2% and the two-step approach was 89.2%. The specificity for *QUIK CHEK* was 98.9%, for Xpert was 96.8%, for BDM was 96.8% and for the two-step approach was 96.2%. The PPV of the *QUIK CHEK* was 90.9%, Xpert was 84.2%, BDM was 84.6% and the two-step approach was 82.5%. The NPV for *QUIK CHEK* was 91.5%, Xpert was 97.3%, BDM was 97.8%, and the two-step approach was 97.2%.

*Clostridioides difficile* toxin EIAs lack sensitivity. All *C. difficile* contain the GDH antigen, whether toxin genes are present or absent. Glutamate dehydrogenase EIAs have high sensitivities but poor specificities; therefore, an additional test (most commonly a toxin assay) must be performed. The GDH EIA is the first test performed in a two-step or three-step approach where a GDH-positive result is followed by a toxin assay or a NAAT to detect toxin genes.^11^ Our findings for the *QUIK CHEK* showed a sensitivity, specificity, PPV and NPV of 54.1%, 98.9%, 90.9% and 91.5%, respectively. These findings were similar to a study conducted in 2012 in Kuwait comparing the Xpert, *QUIK CHEK* and TC, which found the sensitivity, specificity, PPV and NPV of the *QUIK CHEK* to be 53.85%, 100%, 100% and 98.51%, respectively, when using a CRS defined as two tests being in agreement.^19^ A 2009–2018 study conducted by Chung and Lee in Korea compared the diagnostic performance of the *QUIK CHEK* to Xpert as a reference test, with sensitivity, specificity, PPV and NPV of 55.4%, 100.0%, 100.0% and 80.0%, respectively.^20^ However, they had a higher prevalence of *C. difficile* infection (35.9%) than our study population (16.6%), which could explain the difference in PPV (100.0% vs 90.9%).^20^ In contrast, a study conducted in 2013–2014 by Seo et al. in Korea, found a lower sensitivity of 45.7% when using either TC or the combination of *QUIK CHEK* and Xpert as a reference standard.^21^

Nucleic acid amplification tests that target chromosomal toxin genes show high sensitivity and specificity, provide rapid results, and are amenable to both batch and on-demand testing.^22^ Reported estimates of sensitivity for NAATs range from 77% to 100% when compared to TC. Specificity ranges from 83% to 100% in comparison to TC.^23^ nucleic acid amplification tests may detect asymptomatic carriage due to possible non-expression of the toxin encoding gene and therefore the clinical specificity may be lower than reported.^24^ In their meta-analysis conducted in 2019, Kraft et al. reported an estimated sensitivity of 94% and a specificity of 97% when NAATs were compared to either cell culture neutralisation assay or TC or both in studies where it was specifically stated that stools were only included if conforming to the shape of the container.^10^

In this study, the Xpert had a sensitivity, specificity, PPV and NPV of 86.4%, 96.8%, 84.2% and 97.3% respectively, and the BDM performed similarly showing a sensitivity, specificity, PPV and NPV of 89.2%, 96.8%, 84.6% and 97.8%, respectively. A study by Yoo et al. in Korea found a sensitivity of 82.8% for Xpert and 81.6% for BDM when compared to TC. They attributed the difference in sensitivity between the two tests to a freeze-thaw cycle before the BDM testing.^25^ In a study conducted in Germany, Dalpke et al. found a sensitivity, specificity, PPV and NPV of 97.3%, 97.9%, 90.0% and 99.5% for the Xpert and 90.5%, 97.9%, 89.3% and 98.1% for the BDM.^26^

In a study conducted 2014–2017 in South Africa demonstrated the impact of diagnostic methods on the diagnosis of *C. difficile* infection, Nomlomo et al. found a 15.9% positivity rate when using an algorithm approach (consisting of EIA followed by PCR) versus 11.4% and 21.1% when using a toxin EIA and PCR.^27^ However, neither of the two accepted references tests was performed as the comparator test in this study. We showed a 16.6% TC positivity rate which is very similar to Nomlomo et al.’s finding using the algorithm approach.^27^

Similar to the sensitivity of 89.2% and specificity of 96.2% for the two-step algorithm approach in our study, Kraft et al.’s meta-analysis found a sensitivity of 89.0% and specificity of 99.0% when comparing GDH/toxin/NAAT algorithms to the TC or cell culture neutralisation assay.^10^ In contrast, the Seo et al. study found a higher sensitivity, specificity, PPV and NPV of 94.0%, 100.0%, 100.0% and 100.0% for the two-step approach, which could be attributed to the CRS in this study being either TC-positive or a combination of Xpert- and *QUIK CHEK*-positive.^21^ Our algorithm performed similarly to the Xpert or the BDM alone in terms of sensitivity, specificity, PPV and NPV.

The Xpert, BDM and two-step algorithm performed similarly, with overlapping confidence intervals when compared to TC and CRS. From these findings it is evident that TC is a robust reference test for the statistical measures of sensitivity, specificity, PPV and NPV for the Xpert, BDM and two-step approach.

### Limitations

Limitations of our study include the use of stool sampless conforming to the shape of the container as a surrogate for diarrhoea, as well as not excluding other causes of diarrhoea. In addition, we did not collect pre-analytical data such as prior or current antibiotic use or determine any other risk factors for *C. difficile.* Post-analytical patient outcome data was also not collected. The sensitivity of lateral flow assays is limited by the dissociation constant of the antibody–antigen conjugate and by user interpretation of the colorimetric read-out. The TC method used in this study did not include heat-shock treatment prior to the inoculation of samples onto media, which may have improved the detection of *C. difficile*.

### Conclusion

The Xpert and BDM assays and two-step approach performed similarly in detecting toxigenic *C. difficile* in faecal samples. *QUIK CHEK* cannot be used on its own for the diagnosis of *C. difficile* due to its poor sensitivity but the SOC two-step approach using *QUIK CHEK* followed by Xpert showed a similar sensitivity and PPV compared to molecular testing alone. The continued use of the current two-step approach in a resource-limited setting such as South Africa is recommended as it is rapid, easy to perform and reduces cost without compromising diagnostic accuracy.
